# Normalized difference vegetation index as the dominant predicting factor of groundwater recharge in phreatic aquifers: case studies across Iran

**DOI:** 10.1038/s41598-020-74561-4

**Published:** 2020-10-15

**Authors:** Esmaeel Parizi, Seiyed Mossa Hosseini, Behzad Ataie-Ashtiani, Craig T. Simmons

**Affiliations:** 1 Physical Geography Department, University of Tehran, P.O. Box, 14155-6465 Tehran, Iran; 2 Department of Civil Engineering, Sharif University of Technology, P.O. Box 11155-9313, Tehran, Iran; 3 National Centre for Groundwater Research & Training and College of Science & Engineering, Flinders University, GPO Box 2100, Adelaide, South Australia 5001 Australia

**Keywords:** Environmental sciences, Hydrology

## Abstract

The estimation of long-term groundwater recharge rate (${GW}_{r}$) is a pre-requisite for efficient management of groundwater resources, especially for arid and semi-arid regions. Precise estimation of ${GW}_{r}$ is probably the most difficult factor of all measurements in the evaluation of GW resources, particularly in semi-arid regions in which the recharge rate is typically small and/or regions with scarce hydrogeological data. The main objective of this study is to find and assess the predicting factors of ${GW}_{r}$ at an aquifer scale. For this purpose, 325 Iran’s phreatic aquifers (61% of Iran’s aquifers) were selected based on the data availability and the effect of eight predicting factors were assessed on ${GW}_{r}$ estimation. The predicting factors considered include Normalized Difference Vegetation Index (NDVI), mean annual temperature ($T$), the ratio of precipitation to potential evapotranspiration (${P/ET}_{P}$), drainage density (${D}_{d}$), mean annual specific discharge (${Q}_{s}$), Mean Slope ($S$), Soil Moisture (${SM}_{90}$), and population density (${Pop}_{d}$). The local and global Moran’s I index, geographically weighted regression (GWR), and two-step cluster analysis served to support the spatial analysis of the results. The eight predicting factors considered are positively correlated to ${GW}_{r}$ and the NDVI has the greatest influence followed by the $P/{ET}_{P}$ and ${SM}_{90}$. In the regression model, NDVI solely explained 71% of the variation in ${GW}_{r}$, while other drivers have only a minor modification (3.6%). The results of this study provide new insight into the complex interrelationship between ${GW}_{r}$ and vegetation density indicated by the NDVI. The findings of this study can help in better estimation of ${GW}_{r}$ especially for the phreatic aquifers that the hydrogeological ground-data requisite for establishing models are scarce.

## Introduction

Groundwater (GW) is a ubiquitous source of freshwater, which supports human health, socio-economic development and functioning of ecosystems in all climatic regions in developed and developing countries^1, 2^. About 67% of the global groundwater consumption (~ 650 km^3^/year) is extracted in the countries that are characterized by climatic aridity, such as India (30%), USA (17%), Pakistan (10%), China (8.5%), Iran (8.5%), Mexico (4%), and Saudi Arabia (3%)^3^. Motivated by the accessibility of pumping technology, continuing increase in water demands, and along with a decrease in precipitation and surface flows overreliance on GW systems especially in arid and semi-arid regions has led to a groundwater depletion problem^4, 5^. At the same time, aquifer storage replenishment through natural or managed recharge made occurs at a slower rate than its exploitation in such regions^6^. The imbalance between groundwater recharge (${GW}_{r}$) and the combination of natural rates of discharge and anthropogenic GW withdrawal have resulted in the GW continuous diminish in many arid environments^7^ and thus, non-sustainable yield^8, 9^.

In many countries of the Middle East, groundwater resources have insignificant natural recharge, and monitoring the ${GW}_{r}$ rates at which they are utilized under anthropogenic activities is important for sustainable planning purposes^10^. In Iran, the unsustainable rates of groundwater abstraction reaching this country to a point where socio-economic development, political stability, ecosystem integrity, the health and the welfare of natural systems, and human communities are seriously threatened^11^.

Challenges to sustainable yield and efficient management of GW resources are directly linked to the accurate estimation of aquifer system fluxes, especially recharge rate as the key inflow component^12^. Ongoing land-use and land-cover changes from anthropogenic and natural forces could have significant consequences for volume, distribution, and pattern of GW replenishment through natural GW recharge^13^. The estimation of long-term ${GW}_{r}$ rate is a pre-requisite for efficient GW resource management and is difficult to estimate reliably using the traditional methods particularly in semiarid regions which the recharge rate is typically small^14^. Variations and the diffuse nature of ${GW}_{r}$ may also enhance the difficulties of its estimation^15^.

However, the groundwater discharge components (ET, spring-flow, base-flow and pumping) are much more reliably than groundwater recharge to quantify^16^ but, In some countries like Iran, the role of groundwater recharge in water balance equation is more highlighted than GW discharge since the GW withdrawal through pumping wells, springs, and contribution to surface-flow are monitored partially for a few percent of discharge points (usually less than 10%)^17^. The magnitude of ${GW}_{r}$ at a particular location is influenced by five main factors^18–21^: climate (e.g. precipitation, temperature, potential evapotranspiration), soils (e.g. texture, saturated hydraulic conductivity, moisture capacity), hydrology (e.g. streamflow, water table depth), geomorphology (e.g. surface slope, drainage density), land use, land cover (e.g. vegetation density and type). Estimating ${GW}_{r}$ is probably the most difficult factors and the least understood hydrological component in the evaluation of groundwater resources and it is also associated with large uncertainties^18, 22^. Kim and Jackson^21^ and Bekele et al.^14^ reviewed ${GW}_{r}$ estimation methods for phreatic aquifers including groundwater residence time, soil water balance method, soil water flux, inverse modeling, water table fluctuation, groundwater balance, and isotope and tracer profile. No single reliable and comprehensive estimation technique can yet be identified to estimate the aquifer replenishment from the spectrum of those developed^23^. Owing to uncertainties involved in each approach arise from available data, local geographic and topographic conditions, spatial and temporal scale required, Scanlon et al.^24^ suggested using multiple techniques to increase the reliability of the results.

The mathematical models, which rely on a soil–water balance method, may also be used for computing ${GW}_{r}$ when other components of the water balance are well-known^22, 25^. For this purpose, various climate and basin data (land cover, soils groups, geologic data, and topography) is required. This variety of the input data and concomitant various spatial and temporal ranges for both the determination and representativeness of ${GW}_{r}$, complicates the interpretation of the outputs in terms of dominating factors^26^. Much of the earlier research work on the groundwater potential recharge zones and have studied the impact of physical factors as controls of recharge (e.g.^27, 28^), but the effect of vegetation on recharge is less well understood and rarely incorporated^21^. However, evidence for detection of subsurface water reservoir location using a certain vegetation type is reported. In the mid-nineteenth century, Darcy^29^ relates how Father Paramelle—a naturalist who published "The Art of Discovering Springs" in the same year of Darcy’s law—infers the probable presence of subsurface water and even the approximate depth of the water below the ground surface from the nature and strength of the plants^30^. Karaji^31^ in his millennium-old hydrogeology textbook "The Extraction of Hidden Waters" examined how plants indicate the presence of groundwater by studying their roots. One of the indicators pointed out by Karaji^31^ is lush land and the frequency of vegetation and trees, what known as groundwater-dependent ecosystems today^32^.

Vegetation can intercept the rainfall by leaves and branches, and thus, affects the evapotranspiration, and enhance the recharge time into the soil due to the increasing surface storage component^19^. Long-term variations of vegetation indices, such as the normalized difference vegetation index (NDVI), are widely used to characterize the growth cycle of crops (e.g.^33^). NDVI is an index to calculate greenness of vegetation^34^ and is the suitable indicator for determining the long-term changes of vegetation in one zone^35^. This indicator is based on the reflectance of differential which trees, shrubs and plants exhibit for various parts of the radiation spectrum of solar and is calculated by the difference between the near-infrared and visible (red) bands^36, 37^. NDVI values range between − 1.0 and + 1.0, The lowest NDVI value represents non-vegetative cover, while the highest value indicates healthy vegetation^38^.

To the best of our knowledge, only a few studies could be found that investigate the interrelationship of vegetation properties and ${GW}_{r}$. Kim and Jackson^21^ analyzed more than 600 estimates of ${GW}_{r}$, globally and reported that water input (precipitation + irrigation) has the strongest relationship with the ${GW}_{r}$, followed by vegetation type. Singhal and Goyal^39^ obtained a strong polynomial trend of second-order between pre-monsoon NDVI values and ${GW}_{r}$ (with a correlation coefficient of 0.858). In their study, increase in value of NDVI from 0.13 to about 0.18, the estimated value of ${GW}_{r}$ increases. This is expected as at this level of vegetation; water is retained at the surface due to increase in vegetation density and thus has a greater chance of infiltrating into the ground and thus, limits the overland flow rate. However, when the value of NDVI is greater than 0.18, the groundwater recharge starts decreasing with increasing in NDVI value. This would be due to the reason that vegetation density has now increased to such a level that the interception and absorption of rainwater outweigh the factors responsible for further increase in recharge.

In Parmelia aquifer, a deep phreatic aquifer in Western Australia, groundwater levels have risen between up to 55 cm/year over the last three decades due to the replacement of deep-rooted native vegetation with pasture and annual crops^14^. Applying a grid-based water balance model, the spatial ${GW}_{r}$ variation in Ergene river catchment, Turkey is controlled in order of significance by vegetation land-use, soil group types, and climate^23^. The relationships have also been found for NDVI and changes in groundwater levels^40^ and groundwater flow discharge^41^.

The challenges to assess the relationship between vegetation properties (e.g. density and type) and ${GW}_{r}$ that may be due to the cost and time associated with the collection and preparation of ground- or remotely sensed data^42^. During last decade, emerging Google Earth Engine (GEE), a cloud-based geospatial processing platform which enables users to discover, analyze and visualize climate-weather and geophysical big datasets in powerful ways has enabled progress to be made^43^. This free-to-use platform along with the progress in remote sensing and GIS technology can provide a very effective means to map crops, due to their fast response, periodic observations, and low cost^44^.

In this study, we aimed to clarify and emphasize the explanatory power of long-term NDVI as a proxy or characterization of vegetation density for estimating ${GW}_{r}$ in phreatic aquifers. To reveal the importance of NDVI in estimation of ${GW}_{r}$, a range of factors including climatic (precipitation, potential evapotranspiration, and temperature), hydrological (specific discharge), geomorphological (slope and drainage density), human (population density) and soil properties (soil moisture) are considered and their relations with ${GW}_{r}$ are analysed. A quantitative understanding of the extent of changes of surface vegetation and associated impacts on ${GW}_{r}$ is crucial. We use stepwise and geographically weighted regression (GWR) models along with the two-step cluster analysis to identify the main predicting factors of ${GW}_{r}$ in 325 Iran’s phreatic aquifers. This synthesis is, to our knowledge, the first attempt globally to quantify the relative importance of predicting factors (especially vegetation) on ${GW}_{r}$.

## Materials and methods

**Figure 1:**
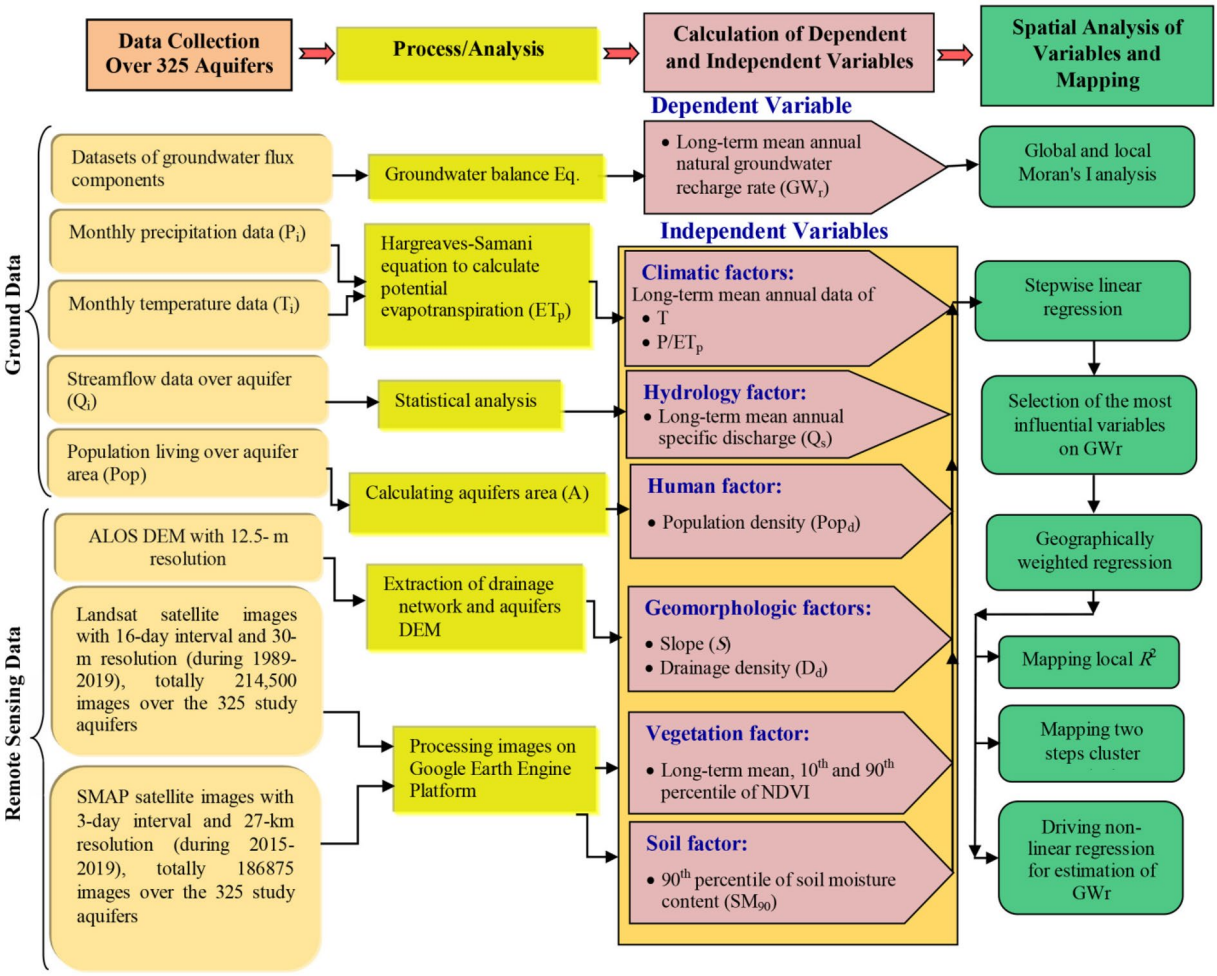
Flowchart of methodology adopted in this study for spatial analysis and estimation of natural groundwater recharge using the considered explanatory factors for 325 Iran’s phreatic aquifers.

Techniques used in this study for spatial analysis of the relationship between ${GW}_{r}$ and predicting factors considered for 325 Iran’s phreatic aquifers are shown schematically in Fig. 1. They include developing stepwise regression, GWR model and cluster analysis to classify hydrologically distinct regions on the degree of impact of each driver on ${GW}_{r}$ estimation. Predicting factors for ${GW}_{r}$ considered in this study include: long-term (30-year during 1989–2019) NDVI as an explanatory proxy or measure of land cover factor; long-term (30-year) mean annual temperature ($T$) and the ratio of precipitation to potential evapotranspiration (${P/ET}_{P}$) as climatic factors; long-term (30-year) mean annual specific discharge (${Q}_{s}$) as a hydrologic factor; mean slope ($S$) and drainage density (${D}_{d}$) as geomorphological factors, soil moisture (${SM}_{90}$) as soil factor and population density (${Pop}_{d}$) as a proxy of urbanization effect.

### Study areas and datasets

**Figure 2:**
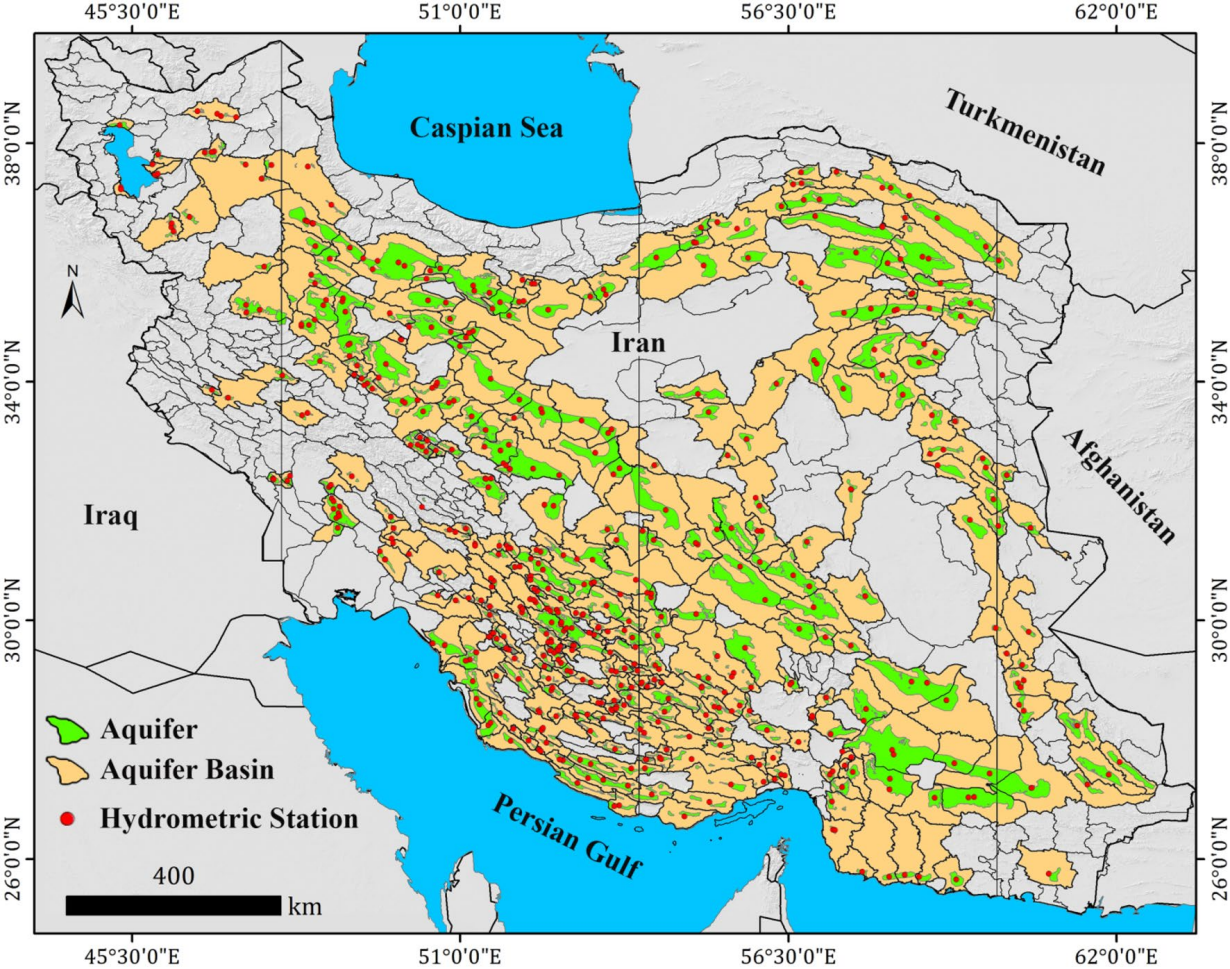
Location of 325 Iran’s phreatic aquifers considered in this study to assess the relationships of explanatory factors and groundwater recharge. The map was generated using ArcGIS Desktop 10.7.1, https://desktop.arcgis.com/en.

Iran’s WRM Co^17^ explored 535 unconsolidated aquifers across the country (494 phreatic aquifers and 41 phreatic-confined aquifers) based on geology and geophysics studies, exploration wells logs, type of sediments, and depth of bedrock investigations. The consolidated aquifers (e.g. karstic aquifers) generally located in the mountainous areas specially Zagros Mountain in west and southwest part of Iran and not considered in this study. Based on the hydrogeological data availability, 325 Iran’s unconfined aquifers (61% of unconsolidated aquifers) were selected and consisted of our research areas as shown in Fig. 2. Moreover, the selected aquifers mostly (89.5%) located in an arid and semi-arid climate, and less in humid (5.3%) and Mediterranean (5.2%) as shown in Fig. 2.

### Natural groundwater recharge estimation

Long-term (30-year) average values of natural recharge for 325 phreatic aquifers across Iran calculated have previously been calculated using a water balance equation by Iran’s Water Resources Management Company^17^. This is considered as a response variable. For this purpose, a lumped water balance model was adopted by Iran’s WRM Company to determine the long-term ${GW}_{r}$ for each aquifer. The total natural groundwater recharge to a phreatic aquifer using the general groundwater balance equation can be defined as^45^

1 $${GW}_{r}={D}_{g}+{S}_{r}+{O}_{g}+\Delta V$$

where ${GW}_{r}$ is total natural groundwater recharge from rainfall, river seepage, return flows from water used for irrigation, domestic, and commercial sectors, and groundwater inflow from other basins; ${D}_{g}$ is draft from groundwater by pumping wells, springs, and qanats;${S}_{r}$ is Groundwater drainage into surface water (e.g. lake and streamflow); ${O}_{g}$ is groundwater outflow to other basins; and $\Delta V$ is the change in groundwater storage.

All components of the water balance equation are computed by Iran’s WRM Co^17^ using independent methods; involve errors due to uncertainties in method’s data required and shortcomings of the techniques used. In many cases, the water balance equation does not balance. The discrepancy of the water balance equation arises from the errors in the calculation of the components and/or components which are not considered is known as residuals term. In Iran, the component of ${GW}_{r}$ is calculated as the unbalanced term (i.e. residual term) of Eq. 1. To reduce the error, long-term averages values of components are considered which generally have smaller errors of estimation than short term averages. According to previous studies (e.g.^46, 47^) the accuracy of water budgets decreases as shorter time frames are considered.

### Calculating NDVI

We used remote sensing data acquired by the Landsat 5 TM and Landsat 7 ETM + satellites as its 30-m resolution can target a fine spatial configuration. This avoids the inclusion of potentially irrigated crops which would have been almost impossible with commonly used larger resolution imagery such as MODIS or AVHRR (with resolutions of 250 m or greater). For calculating the NDVI time-series, 660 Landsat images with 16-day interval and 30-m resolution during 1989–2019 for each aquifer (totally, 214,500 images for 325 aquifers) were processed in Google Earth Engine platform.

Following atmospheric correction, NDVI was calculated using Eq. (2) for all images using bands 3 and 4 in Landsat which have been calibrated to sense radiation in the visible ($Red$) and near-infrared ($NIR$) regions of the spectrum respectively^48^:

2 $$\mathrm{NDVI}=\frac{NIR-Red}{NIR+Red}$$

We used mean, 10th and 90th percentiles of NDVI values for our analysis to assess the effect of low, average and high levels of vegetation coverage of aquifer surface, respectively, on ${GW}_{r}$ estimations. The low, and high vegetation coverage conditions denote vegetation during non-growing and growing season of crops, respectively^22, 49, 50^.

### Calculating other predicting factors of GW recharge

Potential evapotranspiration (${ET}_{P}$) over study aquifers were computed in monthly scale by Hargreaves–Samani Equation^51^. Hargreaves equation is one of the most precise and simplest empirical equations which is used to estimate ${ET}_{P}$ and relies on monthly minimum, maximum, and average temperature and extraterrestrial radiation (${R}_{a}$)^51–54^. This method is more accurate for arid and semi-arid regions and gives reliable results^55–57^.

The ratio of long-term annual average values of precipitation ($P$) over the aquifer area to the ${ET}_{P}$ gives the predicting factor of ${P/ET}_{P}$. long-term mean annual specific discharge (${Q}_{s}$) was calculated using dividing long-term mean annual streamflow ($Q$) by the area of the aquifer ($A$). The Drainage Density (${D}_{d}$) over each study aquifer is obtained by dividing the total length of all the streams over the aquifer area by the area of the aquifer ($A$). The mean slope ($S$) was calculated using DEM and Slope tools in ArcGIS software^58^. Population density (${Pop}_{d}$) is calculated by dividing the total number of peoples living over the aquifer area by the aquifer area ($A$). The long-term soil moisture content in the upper layer (depth of 0–273 mm) of the vadose zone is obtained by SMAP satellite images with 3-day interval and 27-km resolution during 2015–2019, totally 186,875 images over the 325 study aquifer process in Google Earth Engine platform.

By adopting the above factors, we aimed to emphasize the role of surface-motivated predicting factors of ${GW}_{r}$, especially NDVI. Considering other parameters (e.g. hydrogeological properties of aquifer) that may also be correlated to ${GW}_{r}$ are not the primary aim of this study. We believe that relating the ${GW}_{r}$ to the factors which may obtained by the remote sensing techniques (e.g. GEE platform) could be used as a preliminary tool for estimation of ${GW}_{r}$ magnitude, especially in the regions with scares ground-data pre-requisite for model establishing.

### Spatial autocorrelation analysis of ${{\varvec{G}}{\varvec{W}}}_{{\varvec{r}}}$

To investigate the spatial characteristics of ${GW}_{r}$, we used the global and local Moran's I^59^. The global Moran's $I$ (${I}_{G}$) assesses global spatial autocorrelation analysis in the range of [− 1, 1] based on the following formula^60^:

3 $${I}_{G}=\frac{n}{\sum_{i=1}^{n}\sum_{j=1}^{n}{w}_{ij}}\times \frac{\sum_{i=1}^{n}\sum_{j=1}^{n}{w}_{ij}\times \left({x}_{i}-\stackrel{-}{x}\right)\times \left({x}_{j}-\stackrel{-}{x}\right)}{\sum_{j=1}^{n}{\left({x}_{i}-\stackrel{-}{x}\right)}^{2}}$$

where $n$ is the total number of aquifers, ${x}_{i}$ and ${x}_{j}$ are the values of attribute feature of $x$ at location $i$ and $j$, ${w}_{ij}$ is the element of the space weight matrix, $W$ in row $i$th and column $j$th, used to express the neighboring relationship of spatial regions at $n$ location, and $\stackrel{-}{x}$ is the average of all observations for the attribute feature of $x$ in $n$ study areas. This index reflects only the differences in the spatial average. While local Moran's $I$ (${I}_{L}$) examines the distribution pattern of individual attribute values distributed in a heterogeneous space and can measure the degree of local spatial correlation between each area and its surrounding areas^61, 62^:

4 $${I}_{L}={z}_{i}\sum_{j}{w}_{ij}\times {z}_{j}$$

where ${z}_{i}$ and ${z}_{j}$ are the values normalized to regions $i$ and $j$, and ${w}_{ij}$ is an element of the space weight matrix of $W$.

### Stepwise regression model

The stepwise regression model (SRM) is a linear regression that filters independent variables (i.e. predicting factors) that have the most significant influence on the dependent variable (${GW}_{r}$) in a step by step way. When the given explanatory variables are no longer significant, the regression is culled. This process is repeated until all independent variables in the regression are significant^63^.

### Geographically weighted regression (GWR) model

To test the spatial non-stationarity between the most influential explanatory variables (predicting factors) on ${GW}_{r}$ identified by SRM, the GWR model is adopted^64^. The model outputs coefficients of correlation for all aquifers, which are then mapped and tested spatially against raw values to understand what is predicting the most sensitive local relationships^65^. This cartographic approach illustrates the spatial distribution of the sign, magnitude, and significance of the influence of each predicting factor on the dependent variable (${GW}_{r}$). The GWR model reflects the non-stationarity of parameters in different spaces and allows the relationships between variables to change with the spatial position, which provides more realistic results^62^. The formula used by the GWR adopted in this study is the logarithmic transformation of a nonlinear regression as follows^66^:

5 $$\begin{aligned} & \widehat{GW}_{r} = \alpha_{0} \left( {u_{i} ,v_{i} } \right) \times \prod\limits_{k = 1}^{{n_{p} }} {x_{ik}^{{\alpha_{k} \left( {u_{i} ,v_{i} } \right)}} } \mathop{\longrightarrow}\limits{ log }\log \widehat{GW}_{r} = \log \left[ {\alpha_{0} \left( {u_{i} ,v_{i} } \right)} \right] + \alpha_{k} \left( {u_{i} ,v_{i} } \right) \times \sum\limits_{k = 1}^{{n_{p} }} {\log \left[ {x_{ik} } \right]} \\ & x_{ik} = \underbrace {{S,D_{d} }}_{{\begin{array}{*{20}c} {Aquifer } \\ {geomorphy} \\ \end{array} }},\underbrace {{T,P{/}ET_{P} }}_{Climate},\underbrace {{\text{Q}}}_{Hydrology},\underbrace {NDVI}_{{\begin{array}{*{20}c} {Land } \\ {cover} \\ \end{array} }},\underbrace {{Pop_{d} }}_{Human} \\ \end{aligned}$$

where ${\widehat{GW}}_{r}$ is the estimated value of GW recharge as a dependent variable for $i$th aquifer; (*u*_*i*_, *v*_*i*_) are the geographic coordinates for $i$th aquifer; *a*_0_ (*u*_*i*_, *v*_*i*_), and *a*_k_ (*u*_*i*_, *v*_*i*_) are the intercept and local coefficients for $i$th aquifer, respectively; ${n}_{p}$ is the number of predicting factors included in regression (i.e. five variable); ${x}_{ik}$ is the $k$th explanatory variable for $i$th aquifer, and ${\varepsilon }_{i}$ is the random error term for $i$th aquifer. According to the Eq. 5, the logarithm of ${GW}_{r}$ values for 325 aquifers and corresponding five predicting factors considered as GWR inputs. Following the typical estimation method^67^, the regression coefficients for $i$th aquifer are estimated as follows:

6 $${\boldsymbol{\alpha }}_{i}={\left({{\varvec{X}}}^{T}{{\varvec{w}}}_{i}{\varvec{X}}\right)}^{-1}{{\varvec{X}}}^{T}{{\varvec{w}}}_{i}{\widehat{{\varvec{G}}{\varvec{W}}}}_{{\varvec{r}}}$$

where ${\boldsymbol{\alpha }}_{i}$ is the $k\times 1$ vector of regression coefficients for aquifer $i$ with coordinate (*u*_*i*_, *v*_*i*_); ${{\varvec{w}}}_{i}$ is a diagonal matrix $m\times m$ of spatial weights obtained by the weighting functions quantifying the proximities of aquifer $i$ to its $m$ neighborhoods; ${\varvec{X}}$ is the variable matrix $m\times k$; and ${\widehat{{\varvec{G}}{\varvec{W}}}}_{{\varvec{r}}}$ is the vector of estimated value of GW recharge value $k\times 1$. GWR typically employs a kernel weighting function^68^, to allow data points located nearer to the location of interest to have more influence in the regression calculations. For GWR calculations in this study, we used the Gaussian distance-decay based weighting function as follows^69^:

7 $${w}_{Tckj}=\mathrm{exp}\left(-\frac{{d}_{kj}^{2}}{{b}_{Tc}^{2}}\right)$$

where ${w}_{Tkj}$ is the weight of the observation at site $k$ on the observation at site $j$ for ${GW}_{r}$ when the independent variable is $c$; ${d}_{kj}$ is the distance between site $k$ and site $j$, ${b}_{Tc}$ is the kernel bandwidth for ${GW}_{r}$ when the independent variable is $c$, and $exp$ is the exponential function. When the distance is greater than the kernel bandwidth ($d>b$), the weight rapidly approaches zero ($w\to 0$). In this study, the optimal bandwidth was determined by minimizing the corrected Akaike's Information Criterion (AIC) value^70, 71^. All GWR modeling was done using the GWR4 software package version 4.09, which is freely available online^72^. GWR can be used to calculate a set of local regression results including a local parameter estimate, a local R^2^ value, and a local residual for each sampling site^73^.

### Cluster analysis

The GWR model generated a large number of results which provides a challenge for interpretation^74^. Therefore, based on GWR results, a clustering analysis usually served to further scrutinize the results. Two-step cluster method used in this study is a clustering method that determines the optimal number of clusters^75^. through two steps: first, all records are investigated by distance to construct the classification feature tree, while records in the same tree node have high similarity. In the second step, the nodes are classified using the cohesion method and each clustering result is evaluated using an appropriate criterion (i.e. Bayesian information criterion) which yields the final clustering result^62^.

## Results

### Spatial distribution of dependent/independent variables

#### *Groundwater recharge rate, *${GW}_{r}$

**Figure 3:**
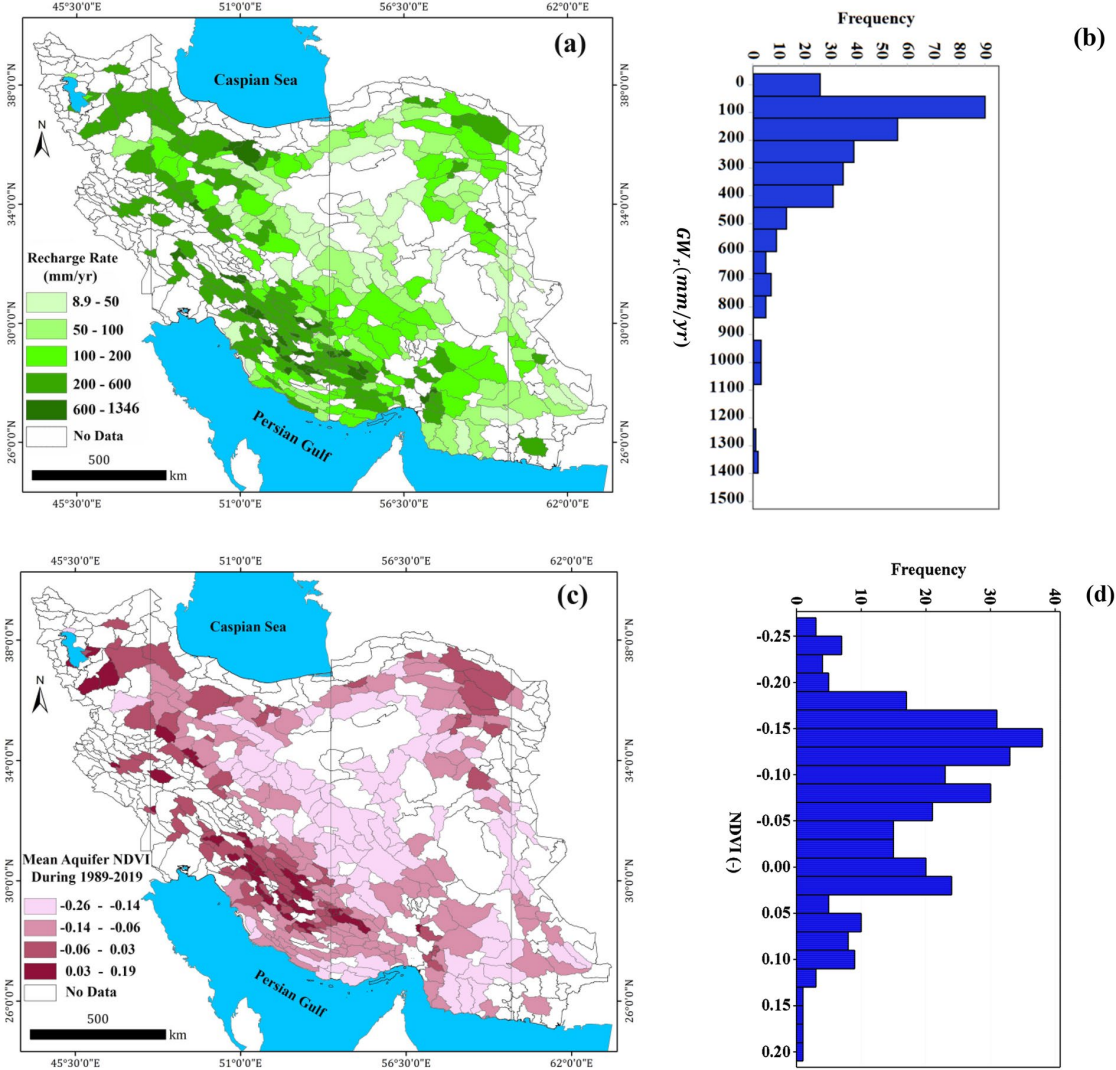

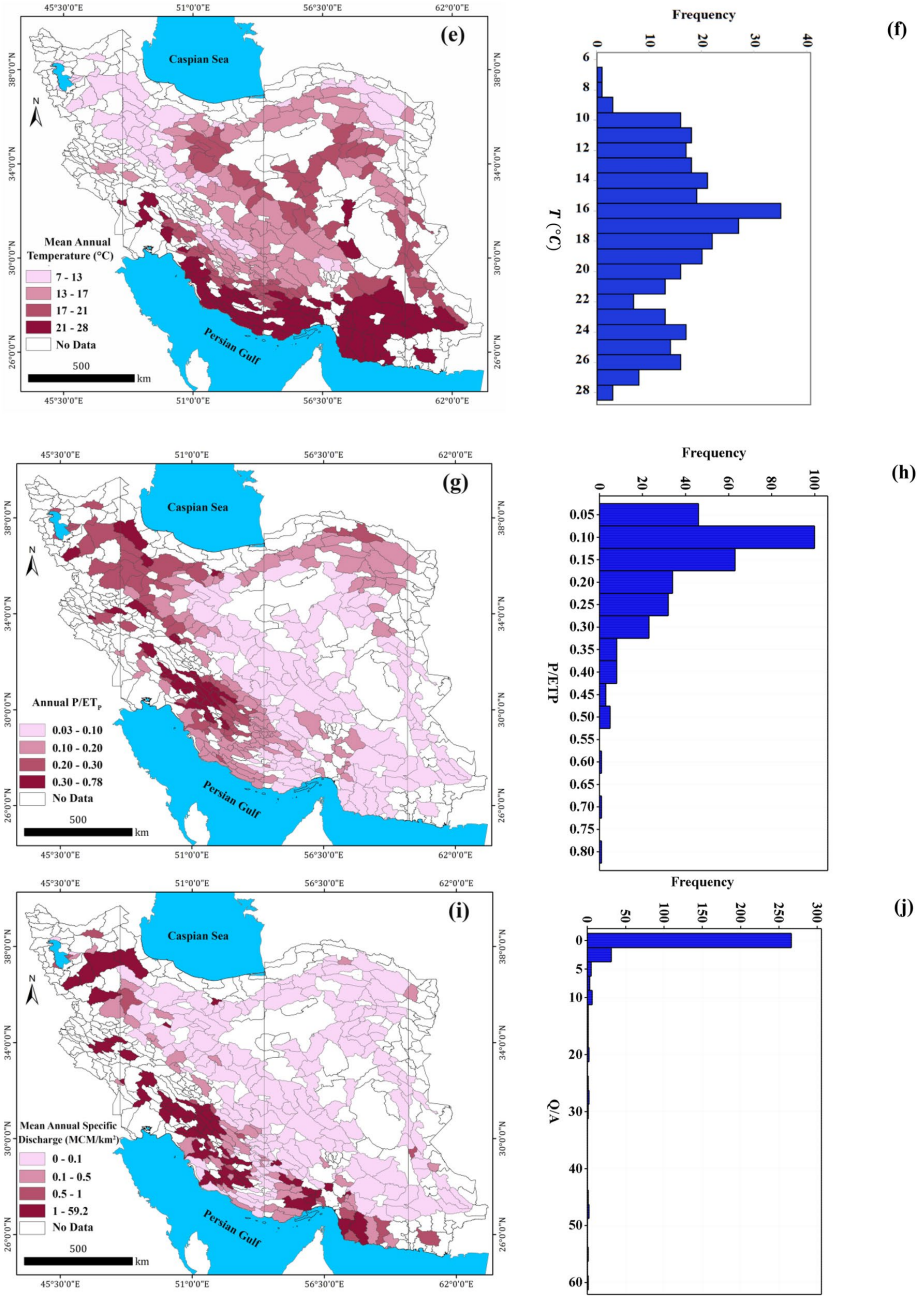

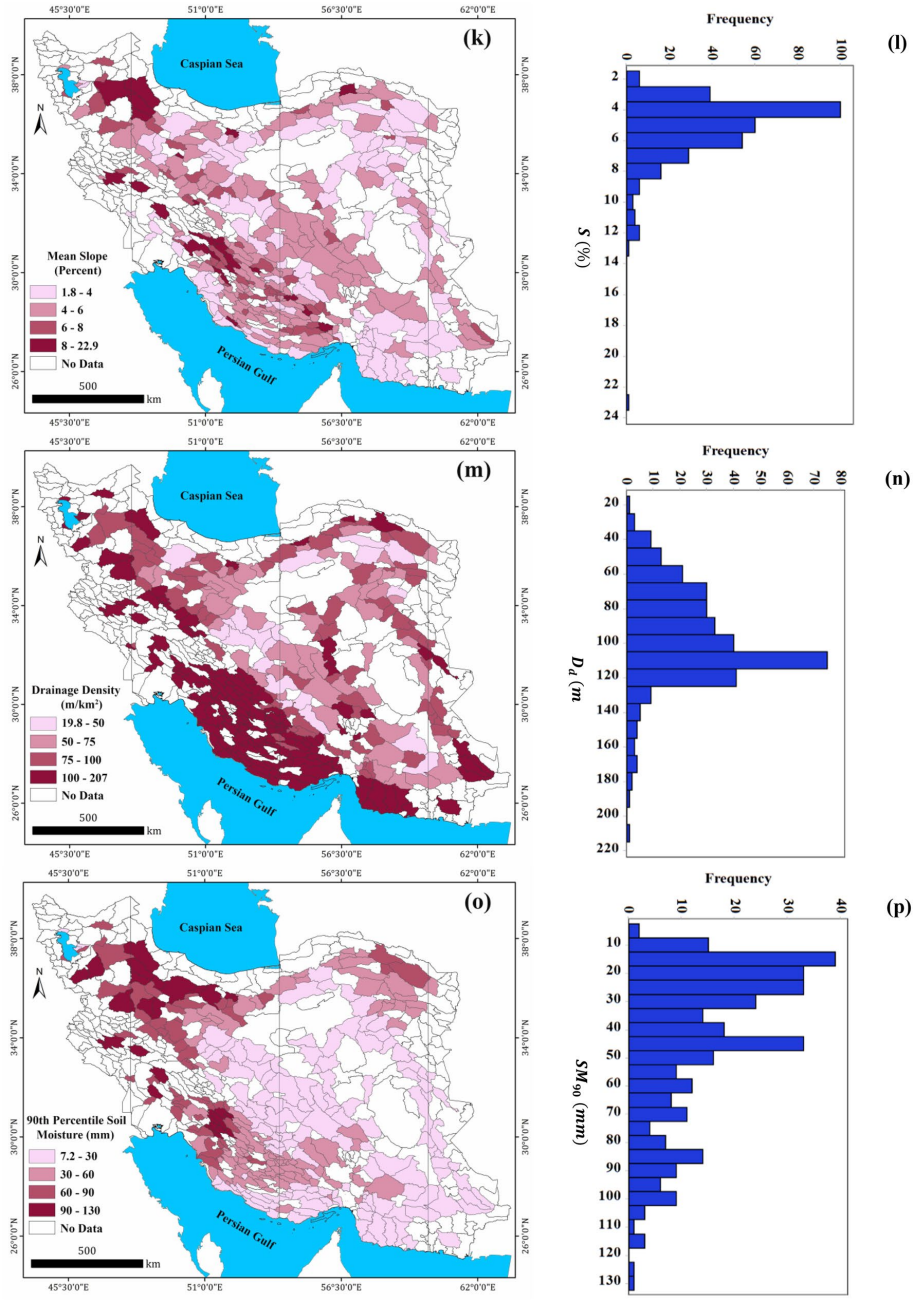

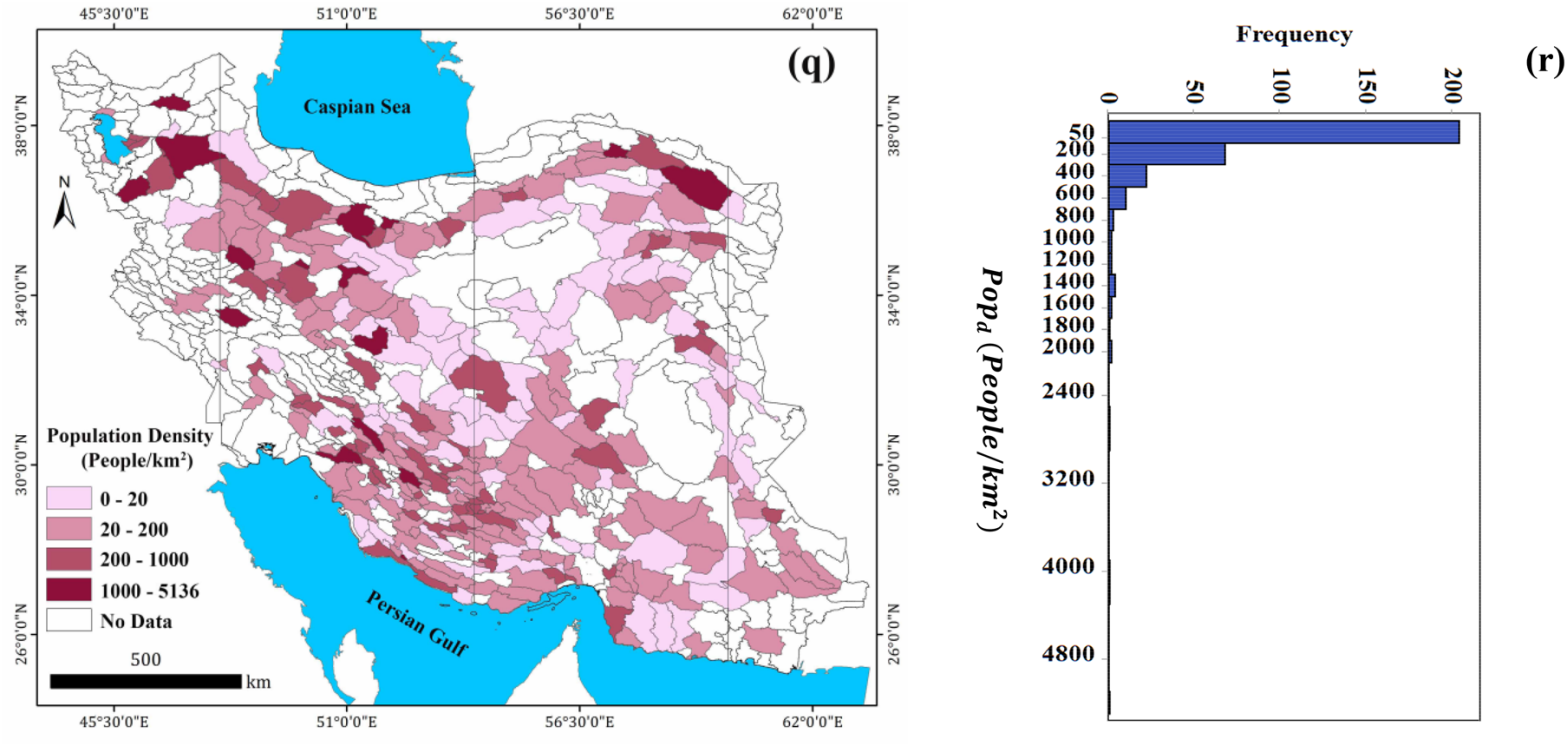
Spatial distribution of long-term mean values of natural groundwater recharge (${GW}_{r}$) (**a**), normalized difference vegetation index (NDVI) (**c**), long-term mean annual temperature ($T$) (**e**), long-term mean annual precipitation to potential evapotranspiration ($P/{ET}_{P}$) (**g**), long-term mean annual specific discharge (${Q}_{s}$) (**i**), mean surface slope ($S$) (**k**), drainage density (${D}_{d}$) (**m**), 90th percentile of soil moisture content (${SM}_{90}$) (**o**), population density (${Pop}_{d}$) (**q**) over 325 Iran’s phreatic aquifers. The corresponding histograms of these layers are also shown (**b**, **d**, **f**, **h**, **j**, **l**, **n**, **p**, **r**). The maps and histograms were generated using ArcGIS Desktop 10.7.1 (https://desktop.arcgis.com/en) and Minitab 16.1 Software (https://www.minitab.com/en-us/products/minitab/), respectively.

The spatial distribution of long-term (~ 30-year) ${GW}_{r}$ values for 325 study phreatic aquifers calculated by Iran’s WRM Company by the year of 2014. The ${GW}_{r}$ is calculated as the sum of recharge from the rainfall, river seepage, return flows from water used for irrigation, domestic, and commercial sectors, and groundwater inflow from other basins as given in the Table S1 in the Supplementary Data. The ${GW}_{r}$ values (as the dependent variable) are in the range of 8.92–1346.8 mm/year (with an average of 257.5 mm/year) as summarized in Table 1. The aquifers with greater ${GW}_{r}$ values are located in southwest, west and northwest of Iran and are associated with the semi-arid, humid and Mediterranean regions (Fig. 2). While 53% of study aquifers receive a recharge rate less than 200 mm/year (Fig. 3b), only 2% of aquifers recharged annually at rates greater than 1000 mm/year mainly located in the southwest region of Iran (due to high precipitation). Noteworthy, Tehran aquifer (located in northern Iran) has received a recharge rate more than 800 mm/year mainly due to leakage from water supply network and sewage network in Tehran city^76^. The histogram of the ${GW}_{r}$ values (Fig. 3b) indicates a positive skewness (1.86) which reveals the higher frequency of the aquifers with a recharge rate less than the mean value.

**Table 1:** Summary of the dependent and predicting variables statistics for 325 Iran’s phreatic aquifers.

Type of Variable	Category	Variable	Description (unit)	Min	Max	Mean	Median	Std. Dev
Dependent	–	${GW}_{r}$	Natural groundwater recharge rate (mm/year)	8.9	1346.8	257.4	182.3	233.8
Predicting (explanatory) variables	Vegetation	$NDVI$	Mean $NDVI$ over aquifers during 1989–2019 (-)	− 0.26	0.19	− 0.08	− 0.09	0.09
	Climate	$T$	Mean annual temperature (°C)	6.9	28.2	17.6	16.8	4.9
		$P$	Mean annual precipitation (mm/year)	45.7	992.5	263.5	232.0	149.2
		${ET}_{P}$	Mean annual potential evapotranspiration (mm/year)	871.1	2464.5	1637.6	1630.3	288.4
	Hydrology	${Q}_{s}$	mean annual specific discharge (MCM/km^2^)	0.0	59.2	2.0	0.08	7.2
	Geomorphology	$S$	Mean slope (%)	1.8	22.9	5.2	4.7	2.2
		${D}_{d}$	Drainage density (m/km^2^)	19.8	207.3	96.7	101.4	28.9
	Soil	${SM}_{90}$	90th percentile of soil moisture content (mm)	7.2	130.4	43.9	38.1	27.9
	Human	${Pop}_{d}$	Population density (people/km^2^)	0.0	5136.9	223.4	57.5	549.9

*MCM* million cubic meter.

#### NDVI as explanation of surface vegetation

As one of predicting factors of GW recharge over phreatic aquifers, the long-term time-series of NDVI values (during 1989–2019 through the processing of Landsat images, totally 214,500) over 325 study aquifers are calculated by GEE cloud platform. A summary statistic of the obtained NDVI values over 325 aquifers are given in Table 1. The NDVI for the study aquifers are in the range of − 0.26 to 0.19 (with an average of − 0.08). Spatial distribution and corresponding histogram of the long-term mean NDVI values are shown in Fig. 3c,d. The time-series of NDVI values for all aquifers are stable over the 30-year period examined. To analyse the effect of low, average, and high levels of vegetation coverage of aquifer surface on the estimation of ${GW}_{r}$, the percentiles of 10th, Mean and 90th of NDVI for the study aquifers were computed and utilized. The percentile of 10th, Mean, and 90th percentiles of NDVI can explain the low, average, and high levels of vegetation coverage of the aquifer surface, respectively^77^.

#### Mean annual temperature, $T$

Long-term mean annual temperatures over study aquifers were also computed by the inverse distance weighted (IDW) method in ARC GIS^58^ based on analysis of monthly data of 3128 synoptic and climatological stations during 1989–2019. The spatial distribution of $T$ values is shown in Fig. 3e. The $T$ values over study aquifers ranged between 6.95 and 28.2 °C, with an average of 17.6 °C (Table 1). The histogram of the T values (Fig. 3f) indicates a weak skewness (0.27).

#### *Precipitation to potential evapotranspiration,*${P/ET}_{P}$

As another predicting factor of ${GW}_{r}$, the ratio of the long-term mean annual precipitation to potential evapotranspiration (calculated by Hargreaves–Samani equation) was considered. For this purpose, the monthly temperature data of 3128 synoptic and climatological stations from 1989 to 2019 and the extraterrestrial radiation (${R}_{a}$) for each aquifer were utilized. The spatial distribution of ${P/ET}_{P}$ over the study aquifers is shown in Fig. 3g with relies on the range of 0.03–0.78. The data of ${P/ET}_{P}$ indicates a positive skewness (0.99) as can be observed in the corresponding histogram in Fig. 3h.

#### *Mean annual specific discharge,*${Q}_{s}$

River base flow is taken as equivalent to the total groundwater recharge of a basin and the system is assumed steady state such that groundwater discharge is assumed to equal to the recharge^78^. In the study areas, due to lack of continuous streamflow data, the annual averaged river-flow ($Q$) divided by the area of the aquifer ($A$) is considered as another predicting factor of ${GW}_{r}$. The spatial distribution and histogram of ${Q}_{s}$ values are shown in Fig. 3i,j. A strong positive skewness is observed for this set of data (9.55).The ${Q}_{s}$ values are in the range of 0.0 (no surface flow) to 59.2 MCM/km^2^ for the aquifers located in southwest Iran (with an average value of 2 MCM/km^2^) according to Table 1.

#### *Mean surface slope, *$S$

Another factor that may have a strong influence on ${GW}_{r}$ for the phreatic aquifers is topography. For this purpose, the average surface slope of study aquifers ($S$) is calculated by using ALOS DEM 12.5 m and Slope tools in ArcGIS software^58^. The obtained values of S are in the range of 1.8–23.0% (with an average value of 5.2%) as shown in Table 1 and also Fig. 3k,l.

#### *Drainage density, *${D}_{d}$

Drainage density of the basin over the aquifer’s boundaries (${D}_{d}$) is also computed and considered as another predicting factor of ${GW}_{r}$. The calculated values of ${D}_{d}$ are in the range of 19.8–207.3 m/km^2^ (with an average of 96.7 m/km^2^) as shown in Fig. 3m,n and also Table 1.

#### *Soil moisture content*, ${SM}_{90}$

The long-term value of soil moisture content ($SM$) in the upper layer of soil (in the depth of 0–273 mm) for the study aquifers are considered as another predicting factor of ${GW}_{r}$. Because ${GW}_{r}$ occurs when the infiltrated water exceeds the maximum soil moisture capacity (${SM}_{max}$), the 90th percentile of daily data of soil moisture content (${SM}_{90}$) could be a good approximation for this threshold value^79^. In deep phreatic aquifers, which is true for the most of Iran’s aquifers (mean depth to water table 34 m), the presence of thick unsaturated zone buffers the water table response to rainfall.

Due to lack of ground-data for $SM$, such data is derived through remote sensing from SMAP satellite. In total, 186,875 images (27-km resolution and 3-day intervals) were analyzed in GEE platform for 325 study aquifers (575 images for each aquifer) during 2015–2019. Using the time-series data of $SM$ over the aquifer area, the 90th percentile of $SM$ data (${SM}_{90}$) for each aquifer were calculated, daily and considered as one of predicting factor of ${GW}_{r}$. The spatial distribution of long-term ${SM}_{90}$ data for the study aquifers are shown in Fig. 3o,p. According to Table 1, the values of ${SM}_{90}$ for the study aquifers are in the range of 7.2–130.5 mm (44.0 mm on average).

#### *Population density,*${Pop}_{d}$

In the urban areas, ${GW}_{r}$ results from rainfall infiltration (across the pervious areas), and leakage from water supply and sewerage networks and thus, varies widely with population density and development level^80^. In this study, the population density (${Pop}_{d}$) over the aquifer areas was considered as an available explanatory variable of urbanization and the human effect on ${GW}_{r}$. The ${Pop}_{d}$ values have a broad range of 0.0 (without habitant) to 5137 people/km^2^ (averagely 224 people/km^2^) as shown in Fig. 3q and Table 1. The data of ${Pop}_{d}$ indicates strong positive skewness (5.45) as can be seen from their corresponding histogram (Fig. 3r).

The correlation matrix of dependent and independent variables based on the initial data shown in Table 2 gives more information about the relation between the ${GW}_{r}$ and the predicting factors. The ${GW}_{r}$ has the maximum correlation with NDVI (0.74), followed by the $P/{ET}_{P}$ (0.46) and ${SM}_{90}$ (0.39). Dense surface vegetation (i.e. higher values of NDVI) may be associated with the potential zones for recharge to underlying layers. While previous studies reported that the groundwater level (GWL) has a strong linear relationship with NDVI, especially for shallow aquifers during dry years (e.g.^40^), a non-significant relationship is observed between NDVI and GWL. This may be due to the depth of GWL in Iran’s aquifers (mean 34 m) are much greater than interacts with surface vegetation. Of note is that the ${GW}_{r}$ has a positive correlation (direct relationship) with all considered predicting factors. The positive correlation of ${GW}_{r}$ and ${Pop}_{d}$ in Table 2 (0.23) can be interpreted with increasing population the rate of return flows of domestic use to groundwater resources increases, especially for the urban areas that the source of domestic water supplies from outside of aquifer basin. Surprisingly, increasing the surface slope of the study aquifers ($S$) increased the ${GW}_{r}$ rate, due to the topography, geomorphological and climatic condition of Iran’s basins. Usually, in Iran, lower slope lands consist of with fine-grain sediments (e.g. clay and silt) which have a low rate of ${GW}_{r}$.

**Table 2:** Cross-correlation matrix of dependent (${GW}_{r}$) and predicting factors (NDVI, $T$, $P/{ET}_{P}$, ${Q}_{s}$, $S$, ${D}_{d}$, ${SM}_{90}$, ${Pop}_{d}$) considered in this study (original data without transformation were considered).

Variable	${GW}_{r}$	$NDVI$	$T$	$P/{ET}_{P}$	${Q}_{s}$	$S$	${D}_{d}$	${SM}_{90}$	${Pop}_{d}$
${GW}_{r}$	1.00								
$NDVI$	0.74***	1.00							
$T$	0.01	− 0.13*	1.00						
$P/{ET}_{P}$	0.46***	0.64***	− 0.38***	1.00					
${Q}_{s}$	0.26**	0.25**	0.11*	0.379***	1.00				
$S$	0.18*	0.35***	− 0.32***	0.48***	0.14*	1.00			
${D}_{d}$	0.21**	0.33***	0.23**	0.34***	0.13*	0.22**	1.00		
${SM}_{90}$	0.39***	0.56***	− 0.43***	0.75***	0.34***	0.40***	0.14*	1.00	
${Pop}_{d}$	0.23**	0.20**	− 0.18*	0.24**	0.09	0.15	0.04	0.26	1.00

${GW}_{r}$ long-term mean annual groundwater recharge, *NDVI* long-term mean annual normalized difference vegetation index, $T$ long-term mean annual temperature, $P/{ET}_{P}$ ratio of long-term mean annual precipitation to potential evapotranspiration, ${Q}_{s}$ mean annual specific discharge , $S$ mean surface slope, ${D}_{d}$ drainage density, ${SM}_{90}$ 90th percentile of soil moisture content, ${Pop}_{d}$: population density.

*Significant at 90% confidence level.

**Significant at 95% confidence level.

***Significant at 99% confidence level.

While $P/{ET}_{P}$ and ${SM}_{90}$ are the strong drivers of NDVI with direct relationship, the temperature has negatively correlated with NDVI (Table 2). This is consistent with other studies which suggested temperature to be negatively correlated with NDVI during spring^81^ and summer^82^. This negative relationship can be due to lower soil moisture caused by higher temperature, especially in the regions with limited rainfall. The importance of evaporation and its negative impact on NDVI has been reported in other studies^81^.

### Global and local Moran’s indicator

**Figure 4:**
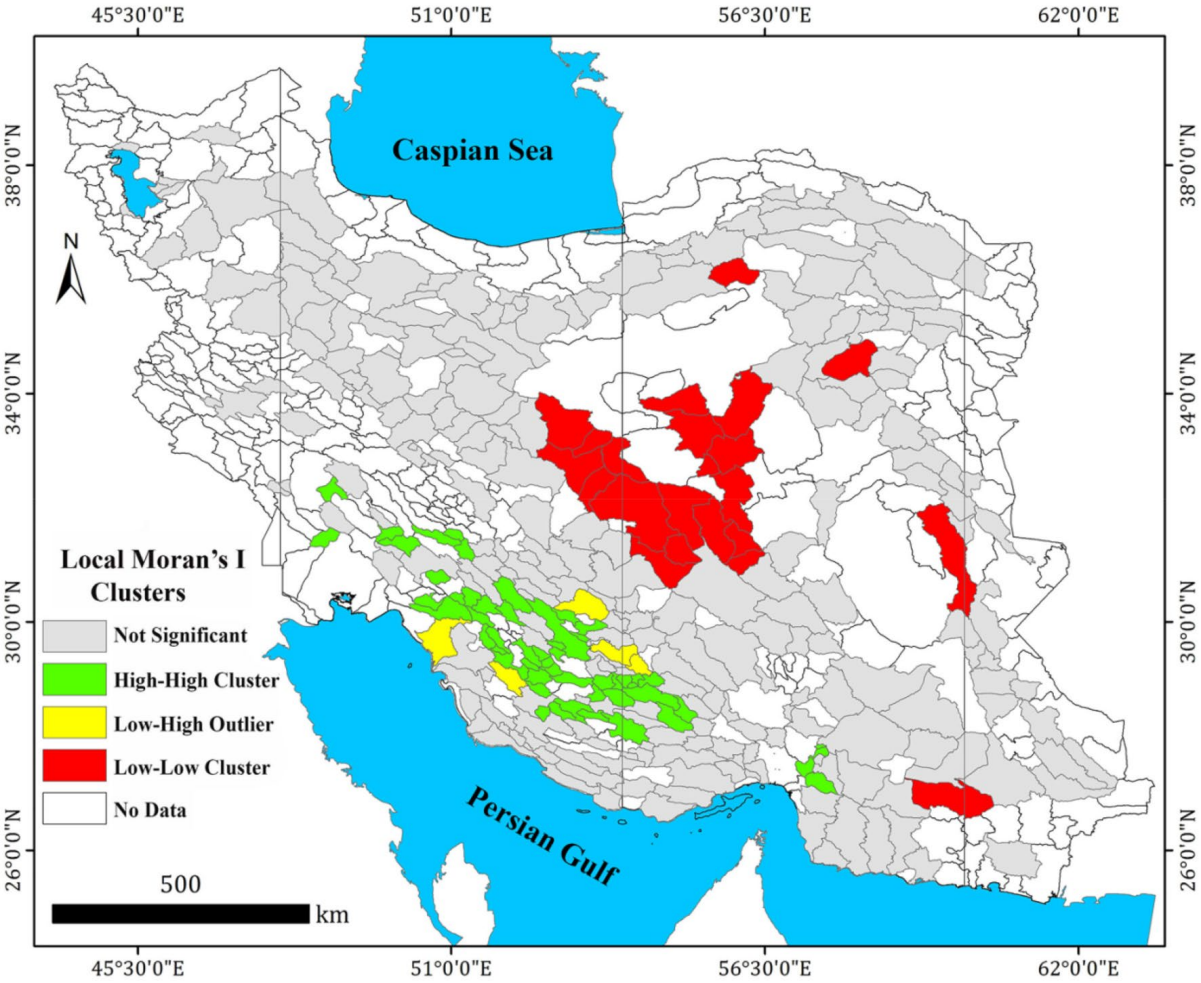
Local Moran's I clusters of groundwater recharge for 325 Iran’s phreatic aquifers. The map was generated using ArcGIS Desktop 10.7.1, https://desktop.arcgis.com/en.

The spatial characteristics of ${GW}_{r}$ in Iran’s phreatic aquifers is investigated by the global and local Moran's I indicator. The global Moran's I for ${GW}_{r}$ data is 0.373 ($p$< 0.01), which indicates an important positive spatial correlation for this variable. To better understand the spatial characteristics and distribution of ${GW}_{r}$ across the study aquifers, the local Moran's I indicator was used. Figure 4 shows the results of Moran's I indicator, locally. There are several aquifers of high-high cluster in southwest Iran (especially Fars Province) which indicates these aquifers have high value of ${GW}_{r}$ and neighboring to such aquifers. One of aquifer included in this cluster is Roudan in Hormozgan Province (southern Iran) with an annual recharge of 826 mm/year. The high value of recharge rate associated with this aquifer leads to Hosseini et al.^11^ ranked it as the third Iran's aquifers from viewpoint of sustainable management. The aquifers which classified as low-low clusters (e.g. ones located in Yazd, Esfahan, Sistan and Baluchestan, Khorasan and Semnan Provinces) have low values of ${GW}_{r}$ and adjacent to such aquifers. Moreover, the aquifers of Neyriz, Tang-e Hana, Qaderabad, Farashband and Bandar Ganaveh in Fars and Bushehr Provinces are classified as high-low clusters. The last cluster indicates that these aquifers have a low value of ${GW}_{r}$ and adjacent to the aquifers with high values of ${GW}_{r}$.

### Stepwise regression (SR) model

The SR model was used to select the most influenced predicting factors of ${GW}_{r}$ in the study aquifers. For this purpose, four criteria of the adjusted coefficient of determination (${R}^{2}$), standard error of estimation ($SE$), Akaike's information criterion ($AIC$), and variance inflation factor ($VIF$) were used and computed for all regressions. As discussed previously, before the variables enter the SR model, the data series of both dependent and independent variables were transformed using the logarithm function. This transformation was used to obtain a constant variance of the residuals about the regression line, and to linearize the relation between the variables to use linear least squares regression techniques. The equations and goodness-of-fit criteria of five types of SR models (SR 1 to SR 5) that includes one (including NDVI with 10th percentile, 90th percentile and mean) to five (NDVI, ${Pop}_{d}$, $T$, ${Q}_{s}$ and ${D}_{d}$) independent variables are shown in Table 3. These regressions are statistically significant at $\alpha \hspace{0.17em}$= 0.01 based on $t$-test values and $F$-test. Since all $t$-test and $F$-values of regressions given in Table 3 are much smaller than 0.01, therefore, the regression results for the selected parameters show the significance of these factors (with a confidence level of 0.99) in explaining the ${GW}_{r}$. Results given in Table 3 indicate that the SR 5 model that includes five independent variables of NDVI (mean values), ${Pop}_{d}$, $T$, ${Q}_{s}$ and ${D}_{d}$ indicates better performance in the estimation of ${GW}_{r}$. The positive exponent of predicting factors in the regressions reveals the direct relationships of these variables on the magnitude of ${GW}_{r}$.

**Table 3:** Results of stepwise regression (SR) analysis and evaluation criteria (all variables are significant at $\alpha \hspace{0.17em}$= 0.01) for estimation of groundwater recharge rate over the study aquifers.

SR no.	Equation	Model evaluation criteria			
		Adjusted $R$^2^	$AIC$	$VIF$	$SE$
SR 1	${GW}_{r}$ = 0.002 × ${\left({NDVI}_{10}\right)}^{2.05}$	0.340	684.61	1.00	0.352
	${GW}_{r}$ = 0.001 × ${\left({NDVI}_{90}\right)}^{3.26}$	0.545	621.18	1.00	0.297
	${GW}_{r}$ = 0.007 × $NDVI$^2.07^	0.711	576.64	1.00	0.243
SR 2	${GW}_{r}$ = 0.012 × $NDVI$^1.94^ × ${Pop}_{d}$^0.05^	0.727	570.04	1.36	0.221
SR 3	${GW}_{r}$ = 0.004 × $NDVI$^1.97^ × ${Pop}_{d}$^0.05^ × $T$^0.32^	0.736	566.20	1.38	0.218
SR 4	${GW}_{r}$ = 0.008 × $NDVI$^1.87^ × ${Pop}_{d}$^0.06^ × $T$^0.26^ × ${Q}_{s}$^0.03^	0.740	557.68	1.82	0.216
SR 5	${GW}_{r}$ = 0.018 × $NDVI$^1.94^ × ${Pop}_{d}$^0.06^ × $T$^0.31^ × ${Q}_{s}$^0.04^ × ${D}_{d}$^0.27^	0.747	551.07	1.95	0.214

The equations given in table are back transformed logarithmically from linear regression.

${GW}_{r}$ natural groundwater recharge rate (mm/year), $NDVI$ mean values of normalized difference vegetation index obtained by Landsat satellite images with interval 16-day and resolution 30-m during 1989–2019, ${NDVI}_{10}$* and *${NDVI}_{90}$ are the 10th and 90th percentile of the NDVI, $T$: mean annual temperature (°C), ${Pop}_{d}$ population density (people/km^2^), ${Q}_{s}$ mean annual specific discharge (MCM/${\mathrm{km}}^{2}$), ${D}_{d}$ drainage density (m/km^2^), $R$^2^ determination coefficient of regression, $AIC$ Akaike information criteria, $VIF$ variance inflation factor, $SE$ standard error of estimations.

It is worth noting that the adjusted ${R}^{2}$ values (in Table 3) associated with the regression models varies between 0.711 for SR1 (including only mean NDVI as an independent variable) to 0.747 for SR5 (including five independent variables). This reveals that the estimation of ${GW}_{r}$ values in the study aquifers using the mean NDVI can solely explain 71% of the ${GW}_{r}$ variations. Adding the four predicting factors of ${Pop}_{d}$, $T$, ${Q}_{s}$ and ${D}_{d}$ will improve the regression efficiency as 3.6% in term of $R$^2^, and 4.5% in terms of $SE$ and $AIC$ criteria. The $VIF$ values calculated for five SR models are less than 2, which indicates that there are not problems of serious multi-collinearity among the independent variables (the $VIF$ value above 5 indicates high correlation that may be problematic).

The effect of 10th, 90th percentiles and mean of NDVI (i.e. explanation of low, high and average levels of vegetation coverage of aquifer surface) on ${GW}_{r}$ in estimations were also investigated in SR modeling. Results given in Table 3 indicate that the mean values of NDVI shows better correlation with ${GW}_{r}$ than 10th and 90th percentiles according to the goodness of fit criteria of $R$^2^, $AIC$, $VIF$, and $SE$. This reveals the pivotal role of average condition of surface vegetation coverage (i.e. mean values of NDVI) in estimation of ${GW}_{r}$ for phreatic aquifers rather than high-level (growing period of crops) and low-level (non-growing period of crops) of vegetation coverage. Thus, the mean values of NDVI better represents the inter-annual surface vegetation variability and its role of the ${GW}_{r}$.

Results shown in Table 3 indicate that the regression cannot explain 29% (for SR1) to 25% (for SR5) of the variations in the ${GW}_{r}$. This may be due to discounting other influential factors that are difficult to quantify (e.g. groundwater inflow/outflow from adjacent basins). Developing a single-variable regression model including NDVI to estimate ${GW}_{r}$ has great promise due to simplicity of the deriving vegetation related index from GEE platform especially for the aquifers with scarce ground data. The five drivers of ${GW}_{r}$ selected by the SR model (i.e. SR 5) are considered as GWR model inputs to obtain the locally-varying relationships between the variables (five predictors and ${GW}_{r}$). Since the SR model uses the multi-collinearity diagnostic test, thus, the variables of ${SM}_{90}$, $P/{ET}_{P}$, and $S$ are excluded from the regressions since they are maximum correlated to the NDVI in the study aquifers.

### GWR model

Spatial analysis of the relationship between ${GW}_{r}$ and five predicting factors (NDVI, ${Pop}_{d}$, $T$, ${Q}_{s}$ and ${D}_{d}$) through GWR model were performed for the study aquifers. Table 4 shows the descriptive statistics of the coefficients. According to Table 4, NDVI and Temperature were the variables with the greatest and lowest spatial coefficients (mean 0.889 and 0.092, respectively) and they can predict ${GW}_{r}$ with a direct relationship.

**Table 4:** The coefficients for the GWR model obtained with considering five explanatory variables (stepwise regression SR5 in Table 3) for estimation of groundwater recharge rate over the study aquifers.

Variable	Maximum	Minimum	Mean	St. Dev
NDVI	1.023	0.673	0.889	0.074
Drainage density (${D}_{d}$)	0.349	0.000	0.160	0.086
Population density (${Pop}_{d}$)	0.163	0.053	0.098	0.027
Specific discharge (${Q}_{s}$)	0.361	0.000	0.097	0.102
Temperature ($T$)	0.125	0.017	0.092	0.022

**Figure 5:**
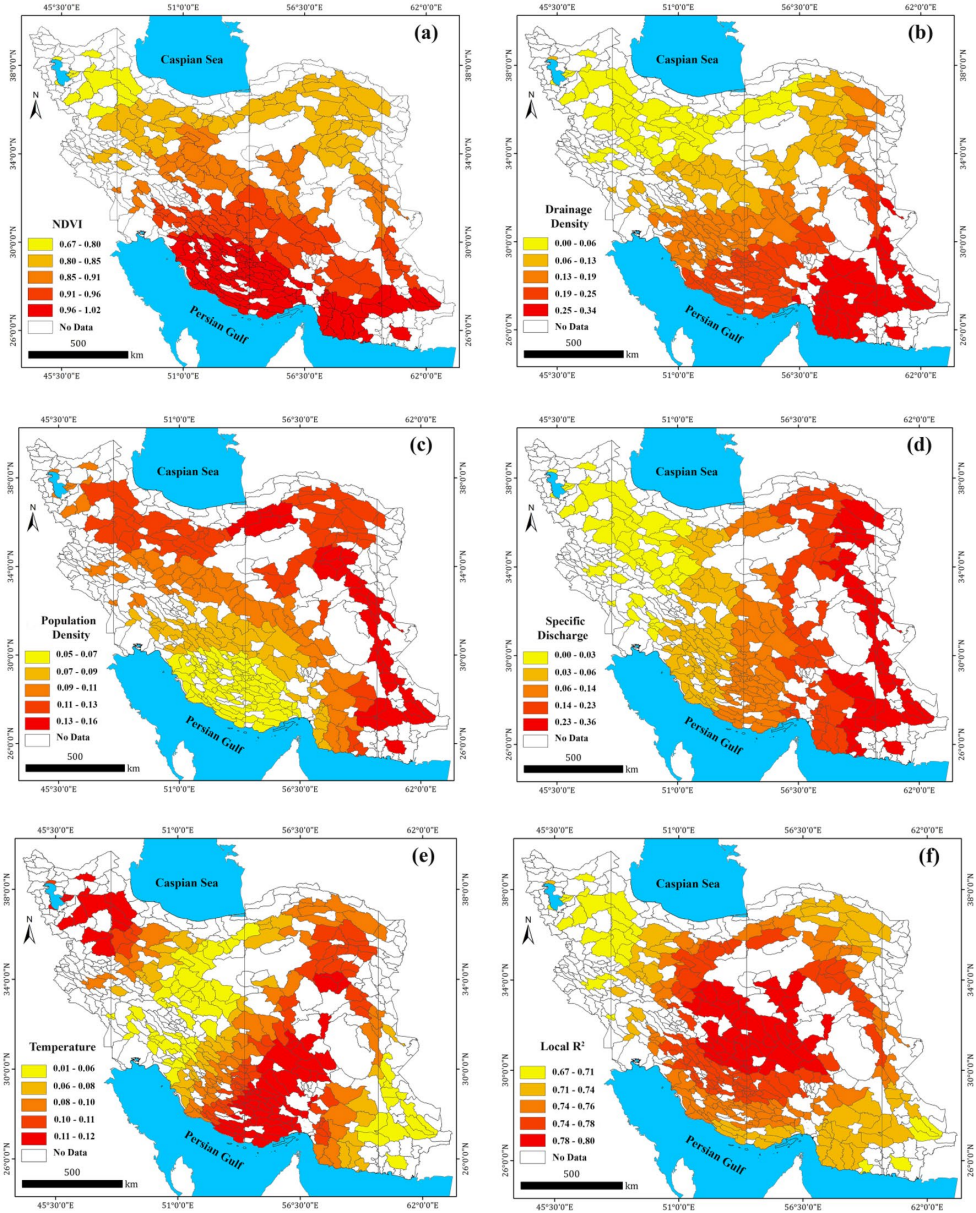
The spatial coefficient distribution for five predicting factors of groundwater recharge over 325 Iran’s phreatic aquifers including average long-term of NDVI, drainage density, population density, specific discharge and annual temperature based on the GWR model (**a**–**e**), and the local $R$^2^ values of the corresponding GWR model (**f**). The maps were generated using ArcGIS Desktop 10.7.1, https://desktop.arcgis.com/en.

In Fig. 5a–e, the coefficients of independent variables were divided into five classes based on the Natural Breaks method^58^. According to Fig. 5a–e, the coefficient of NDVI ranges from 0.67 to 1.02. Significant relationships between NDVI and ${GW}_{r}$ occur in the southwest and south of Iran. The ${GW}_{r}$ for the aquifer located in southern part is highly affected by NDVI. The coefficient for the drainage density (${D}_{d}$) ranges from 0.00 to 0.34 and its coefficient gradually increases from the northwest to the southeast of Iran. The aquifers that are most affected by ${D}_{d}$ are ones located in southeast part. The coefficient for the ${Pop}_{d}$ ranges from 0.05 to 0.16 and the significant relationships are observed in the aquifers in southeast and east parts of Iran. According to spatial distribution of ${Pop}_{d}$ coefficients, the aquifers located in eastern Iran are highly affected by it/them. The coefficient of ${Q}_{s}$ ranges from 0.0 (for west aquifers) to 0.361 (for east aquifers) and its coefficient gradually decrease from the east to the west of Iran. The coefficient of temperature ($T$) ranges from 0.01 to 0.12, and the highest values are observed for the northwest aquifers and the lowest for the southwest regions of Iran. The aquifer which is mostly affected by $T$ is ones located in the northwest part.

The spatial distribution of ${R}^{2}$ values of GWR model is shown in Fig. 5f. The local ${R}^{2}$ value ranges from 0.67 (for northwest aquifers) to 0.80 (for central aquifer). The study aquifers show diversity in degrees of fit (high variation of ${R}^{2}$). Based on Fig. 5f, the local ${R}^{2}$ value increases when moving towards central aquifers of Iran. In other words, The GWR model has the best fit in the central Iran. These results indicate that in the aquifers in the central parts of Iran, the relationship between predicting factors and ${GW}_{r}$ is better in the regression model.

### Cluster analysis of Iran’s aquifers

**Figure 6:**
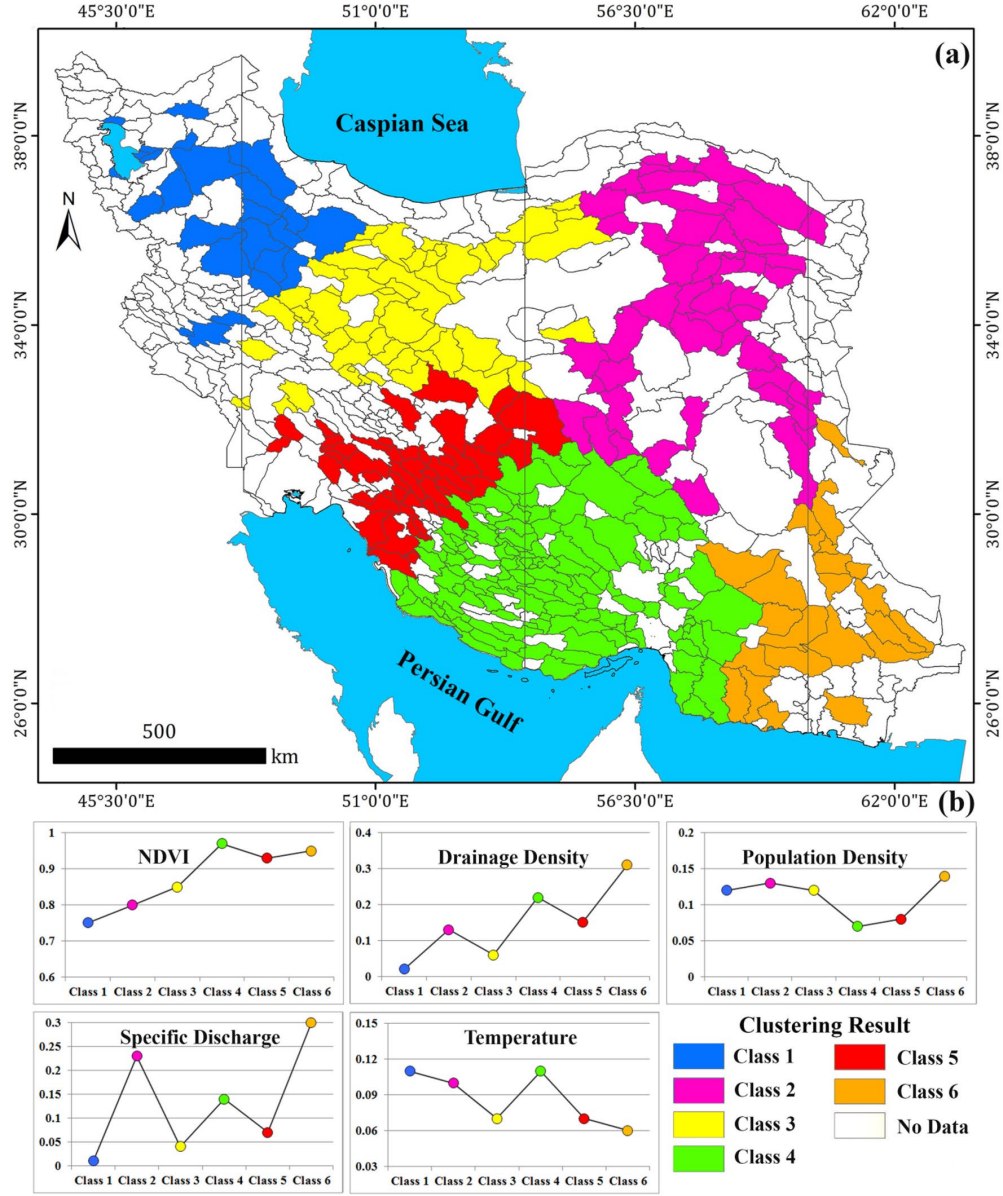
Six types of phreatic aquifers obtained by clustering of the geographically weighted regression coefficients: the distribution of clustering results (**a**) and the clustering results for the GWR coefficients (**b**). The map was generated using ArcGIS Desktop 10.7.1, https://desktop.arcgis.com/en.

Understanding the effects of predicting factors on ${GW}_{r}$ estimation can be obtained by spatial analysis of the GWR’s coefficient through two-step cluster analysis. For this purpose, the study aquifers were divided automatically into six categories based on the Bayesian Information Criterion (BIC) method. The statistical and mapping result of the five predicting factors in each cluster is given in Table 5 and Fig. 6a,b. The coefficients with larger values have a greater impact on the prediction of ${GW}_{r}$. The results shown in Fig. 6 provide valuable information about the recharge predicting factor(s) in each aquifer system.

**Table 5:** Clustering results for GWR model coefficients.

Class	NDVI	Drainage density (${D}_{d}$)	Population density (${Pop}_{d}$)	Specific discharge (${Q}_{s}$)	Temperature ($T$)
1	0.75	0.02	0.12	0.01	0.11
2	0.80	0.13	0.13	0.23	0.10
3	0.85	0.06	0.12	0.04	0.07
4	0.97	0.22	0.07	0.14	0.11
5	0.93	0.15	0.08	0.07	0.07
6	0.95	0.31	0.14	0.30	0.06

Two-step clustering results reveal that the NDVI (as the most influential predictor of ${GW}_{r}$) has the highest effect on the estimation of ${GW}_{r}$ for all classes of aquifers (Table 5). Figure 6 reveals that the effect of NDVI for the aquifers located in south and southwest Iran (aquifers categorized in class 4) is higher than other parts of Iran (the attribute value of NDVI for class 4 is 0.97). These aquifers are characterized by high NDVI and the significant ${GW}_{r}$ (mean 310 mm/year). The lowest effect of NDVI are for the aquifers located in northwest part (aquifers in class 1) which are characterized by NDVI value 0.75 (Table 5). Drainage density, ${D}_{d}$ (as the second most influential predictor of ${GW}_{r}$) has the most effect on aquifers located in southeast of Iran (class 6) which characterized by drainage density > 90 m/km^2^. Population density, ${Pop}_{d}$ (as the third most influential predictor of ${GW}_{r}$) has the highest effect in increasing of ${GW}_{r}$ for the aquifers located in southeast, east, northeast and north of Iran (aquifer of classes 6, 3 and 2). The presence of population on aquifer surface has the lowest effect on ${GW}_{r}$ for the aquifers located in south and southwest parts (class 4). Specific Discharge, ${Q}_{s}$ (as the fourth most influential predictor) has the highest effect on the aquifers located in southeast and northeast of Iran which characterized by mean annual specific discharge 18 MCM/km^2^ (aquifers of classes 6 and 2). Mean annual temperature, $T$ (as the least influential predictor of ${GW}_{r}$) has the most effect on aquifers located in south, southwest and northwest of Iran (classes 4 and 1) which are characterized by mean annual temperature 7–28 °C.

## Conclusion

In this study, the effects of different explanatory variables of climate ($T$, $P/E{T}_{P}$), geomorphologic ($S$ and ${D}_{d}$), hydrologic (${Q}_{s}$), soil (${SM}_{90}$), human (${Pop}_{d}$), and land cover (as NDVI) were analyzed for explaining groundwater recharge rate (${GW}_{r}$) for 325 of Iran’s phreatic aquifers. Of these variables, the stepwise regression consistently indicates the predominant effects of NDVI, ${Pop}_{d}$, $T$, ${Q}_{s}$ and ${D}_{d}$ on ${GW}_{r}$ in the study aquifers. All these predictors are positively correlated with the ${GW}_{r}$. To support the spatial analysis of the results, local and global Moran’s I index, GWR model, and two-step cluster analysis were employed. Results indicated that NDVI is consistently the dominant predictor of ${GW}_{r}$, and followed by the $P/{ET}_{P}$ and ${SM}_{90}$. Thus, land cover is the dominant control on groundwater recharge in all studies areas of Iran.

A consistent and robust story has emerged in terms of the relationships between the predicting factors, especially NDVI, and ${GW}_{r}$ for the phreatic aquifers used as case studies here. Remotely sensed NDVI has allowed rapid collection of data not only across sizeable aquifers, but more importantly, across a time span of years.

In this way, the use of remote sensing along with the GEE cloud platform can be viewed as a strength to provide a large number of hydrological data points for the wide spatial and temporal scales. However, the lack of field studies to verify remotely-sensed observations (NDVI and $SM$) with ground truthed data is a limitation of this study. The results indicate that combining a geographically weighted regression model with two-step cluster analysis can be a valuable tool for identifying the spatial heterogeneity of ${GW}_{r}$ predictors.

In conclusion, relating the remotely sensed data (e.g. NDVI) with ${GW}_{r}$ in the phreatic aquifers will help land-use decisions for sustainable groundwater management, especially where the field data for precise calculation of ${GW}_{r}$ through traditional models does not exist. Among the explanatory variables we investigated, population density (${Pop}_{d}$) and surface vegetation (NDVI) are of manageable ones through human intervention on aquifer surface that are directly related to ${GW}_{r}$ magnitude and its spatial pattern.

## Supplementary information

Supplementary Table S1.
